# Ultrasonic Power Output Measurement by Pulsed Radiation Pressure

**DOI:** 10.6028/jres.101.064

**Published:** 1996

**Authors:** Steven E. Fick, Franklin R. Breckenridge

**Affiliations:** National Institute of Standards and Technology, Gaithersburg, MD 20899-0001

**Keywords:** medical ultrasonics, ultrasonic radiation pressure, ultrasonic transducer calibration, ultrasound power measurements

## Abstract

Direct measurements of time-averaged spatially integrated output power radiated into reflectionless water loads can be made with high accuracy using techniques which exploit the radiation pressure exerted by sound on all objects in its path. With an absorptive target arranged to intercept the entirety of an ultrasound beam, total beam power can be determined as accurately as the radiation force induced on the target can be measured in isolation from confounding forces due to buoyancy, streaming, surface tension, and vibration. Pulse modulation of the incident ultrasound at a frequency well above those characteristics of confounding phenomena provides the desired isolation and other significant advantages in the operation of the radiation force balance (RFB) constructed in 1974. Equipped with purpose-built transducers and electronics, the RFB is adjusted to equate the radiation force and a counterforce generated by an actuator calibrated against reference masses using direct current as the transfer variable. Improvements made during its one overhaul in 1988 have nearly halved its overall measurement uncertainty and extended the capabilities of the RFB to include measuring the output of ultrasonic systems with arbitrary pulse waveforms.

## 1. The NIST Radiation Force Balance—Introduction

Radiation pressure has been the subject of both extensive theoretical study [1–11] and widespread practical use [11–22] in the 90 years that have elapsed since the concept was first advanced [1]. Thorough consideration of its various details has absorbed substantial effort over a wide base of disciplines. Despite its complexity, radiation pressure can be exploited successfully in ultrasound power meters whose principal design equation could not be simpler. An appropriately constructed target properly aligned in a steady-state underwater ultrasound field is subjected to a radiation force *F* given by

F=W/c, (1)

where *W* is the time-averaged spatially integrated power intercepted by the target, and *c* is the speed of sound in the water. Time-averaging of the ultrasound occurs because, under practical circumstances, the inertia of the target causes it to effectively integrate pulses into the corresponding steady state force. Spatial integration is a consequence of the extended geometry of all realizable targets and is exploited by using targets larger in cross section than the incident beam.

Like all modern ultrasound power meters which make use of radiation pressure, the RFB uses time averaging to mitigate deleterious environmental effects. It is the time scale of this averaging, and the way in which the time scale is established, which distinguishes the RFB from other instruments. Instead of using a microprocessor to numerically average the results of repeated measurements with ultrasound present, and perhaps to make use of the statistical variations observed for a similar set of measurements made with the ultrasound absent, the RFB mechanically averages its response to radiation pressure before an electrical signal is generated. Because the nonlinearities of a sensor cannot be counteracted by averaging its output, integrating the first, and most critical, stage of averaging into the mechanism itself constitutes a fundamental improvement. Although mechanical means could perhaps be devised to effect the long term averaging needed for measurements of the steady state radiation pressure, the RFB is designed to average instead a periodic component induced by modulating the ultrasound source with a 50 % duty factor square wave. With the period of this square wave set equal to the period of the simple harmonic motion of the elastically suspended target, the target is made to function as a tuned detector of radiation pressure.

Measurement errors consequent to the imperfect operation of force sensors were precluded by designing the RFB to operate as a force comparator without directly measuring force. This is done by using an actuator to generate a counterforce which, adjusted in magnitude and phase by a human operator, minimizes the velocity of the target as indicated by the highly amplified output of a moving coil sensor. Because its output is used only to determine that motion of the target has been arrested, the velocity sensor need only be linear for small displacements, stable over short time intervals, and need not be calibrated. Such requirements are easily and very conservatively met. Prior calibration of the actuator against reference masses allows levels of force to be inferred from measurements of the dc voltage which controls the actuator.

## 2. Design Details—Mechanical

**Fig. 1:**
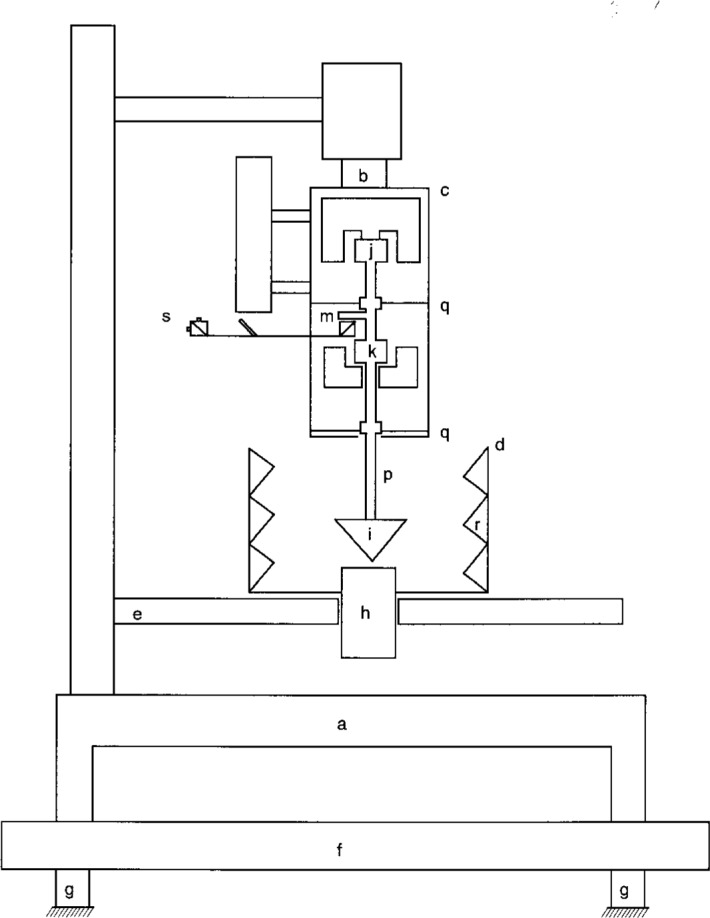
Mechanical elements of the RFB.
a. Drill press frameb. Shaftc. Sensor/driver assemblyd. Test tanke. Drill press tablef. Steel slabg. Pneumatic isolators (2 of 4 shown)h. Transducer of device under testi. Targetj. Velocity sensor coilk. Actuator coilm. Moveable mirror of interferometern., o. Not used as designatorsp. Armature shaftq. Spider assembly (2 of 2 shown)r. Rubber wedges lining wall of test tanks. Interferometer detector assembly

A massive cast iron drill press frame supports the major elements of the RFB, shown in Fig. 1. With its spindle replaced by a shaft from which the mechanical sensor/driver assembly of the RFB is suspended, the drill press head serves as a rigid mount capable of fine adjustment in the vertical position. Coarse adjustment of vertical position, needed during setup of the RFB, is done by moving the drill press table.

Direct transmission of building and room vibrations to the RFB is reduced by its placement on a 100 kg steel slab supported on its bench by four pneumatic isolators. Protection from airborne noise is afforded by placement of the entire apparatus in a sound isolated room equipped with ventilation and air conditioning systems capable of complete stoppage of all forced air circulation. Necessary only for highly sensitive operations, this last feature allows the noise floor for the measurement of highly stable sources to be limited mainly by impulsive low frequency vibrations attributed to vehicular traffic and, therefore, to be controlled by choosing the time of day at which measurements are made.

Ultrasound from the device under test enters the water of the test tank from below and impinges on the target, which constitutes the lower end of the armature of the sensor/driver assembly. Also embodied in the armature, which is the only moving part of the apparatus, are a velocity sensor coil, an actuator coil, and the movable mirror of a Michelson interferometer. These parts are rigidly connected together and constrained to move with only one degree of freedom, in the vertical direction. Motion of the armature is induced by both the radiation pressure applied to the target and by an opposing force generated electromagnetically by the actuator coil. Adjustment of the nulling force to arrest the motion of the armature, as indicated by the output of the velocity sensor, achieves equivalence of the radiation force and the nulling force.

All parts of the armature are carried by a vertical, thin-walled, stainless steel tube which is constrained to vertical rectilinear motion by two spider assemblies each consisting of three flexure wires oriented 120° to each other. Provision for individual adjustment of the tension in each of the six spider wires allows the two armature coils to be centered in their respective magnet gaps. By varying the total tension on the six wires, the stiffness of the suspension, and consequently the armature resonance frequency, may be adjusted. In the absence of other constraints, a resonance frequency on the order of a few hundred hertz, well removed from the lower frequencies typical of vibration due to moving objects, would be chosen to avoid the vibration frequencies typical of line-powered electromagnetic equipment. For the RFB, considerations involving dynamic flexure of the frame supporting the spider assemblies imposed an upper limit on the stiffness of the suspension and, therefore, on the resonance frequency. Calculations, based on measured values of suspension stiffness (3.8 × 10^3^ N/m) and armature mass (69 g), predict a 37 Hz free resonance frequency. With the target immersed in its water bath, which loads the armature, the resonance frequency is approximately 20 Hz, which is low enough to avoid flexural resonances of the RFB frame, and vibrations induced by 60 Hz mains-powered equipment.

**Fig. 2:**
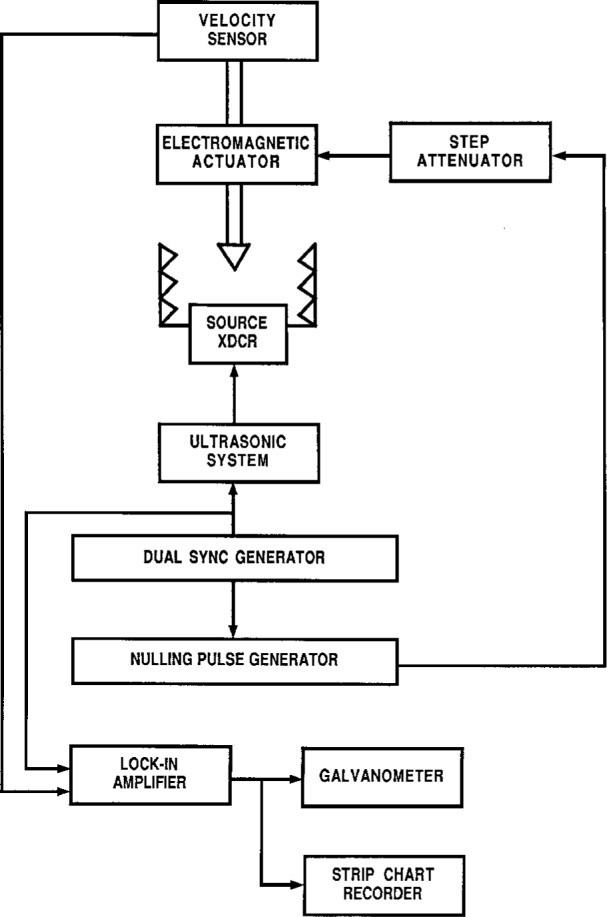
Principal electrical and electronic elements of the RFB.

Designed to minimize radiation force from reflected ultrasound, the target comprises a conical shell of silicone rubber 10 mm thick (measured axially) surrounding a solid cone of rigid polystyrene foam, the whole being 52 mm in diameter (Fig. 2). Both inner and outer conical surfaces have an angle of 45° to their common vertical axis. Ultrasound from the source is directed vertically upward against the vertex of the conical target. An ultrasound ray arriving parallel to the axis of the cone is reflected only slightly at the water-rubber interface because the rubber offers a good acoustical impedance match to that of water. In passing through the rubber, most of the ultrasound energy is absorbed. Near total reflection occurs at the interface between the rubber and the polystyrene foam, since the low density of the foam causes its behavior to approximate that of air, inducing a large mismatch in acoustical impedances. This reflected ultrasound is further attenuated by absorption as it travels through the rubber layer. Because of its radial direction, the reflected ultrasound can only impart to the target radiation forces that are perpendicular to its axis and to which the instrument is insensitive. Furthermore, if the ultrasound beam is radially symmetric about the target axis, the total radiation force from reflected sound is zero.

Compared to traditional metal-shelled reflective conical targets, the absorptive target used with the RFB offers several advantages. One is the ease with which its performance can be measured. Pulse-echo tests show reflected power levels indistinguishable from the 0.1 % noise level of the conventional pulse-echo equipment used for this test. Because the large-signal nonlinearity of such equipment approaches several percent, similar precision in measuring power levels typical of reflective targets could be achieved only with some difficulty. An additional advantage of the absorptive target is its freedom from modal resonances which usually determine the lower operating frequency limit for metal targets. No such resonances have been found for frequencies as low as 0.5 MHz, the lowest frequency for which tests have been requested.

Made from a glass crystallizing dish 190 mm in diameter by 100 mm tall with a circular hole cut in the center of the bottom, the tank rests on the drill-press table, which has a centered hole 88 mm in diameter to allow a transducer and/or its connecting cable to underhang the table. Each transducer to be tested is fitted to the tank by means of a polystyrene-foam block bonded to the tank bottom with silicone rubber adhesive, and carved to accommodate the transducer shape. Installation and removal of the transducer are facilitated by attaching it to the block using electrical tape and duct putty arranged to form a watertight seal. During installation, the transducer is positioned so that its output face protrudes several millimeters into the tank, to ensure that any radiated edge waves will not impinge on the bottom surface of the tank. At the cost of a few minutes of additional labor each time a transducer is installed, this method allows the active surface of the source to be directly coupled to the water, with no membrane or other obstructions in the ultrasound path.

Although the target of the RFB absorbs most of the ultrasound, there is a small amount reflected radially outward toward the cylindrical wall of the test tank. This wall is totally lined with acoustical wedges made of an eraser-rubber material which was found to be a good absorber. These wedges are approximately 20 mm by 25 mm at the base, approximately 35 mm long, and extend radially inward from the cylindrical wall. Tests of the absorber bank in tandem with a reflective 45° conical metal target indicated back reflected power levels no greater than 3 %.

## 3. Electrical Design Details—General

A block diagram of the major electronic systems of the RFB is shown in Fig. 2. Most of these systems are part of a compact purpose-built package composed of inexpensive, easily obtained small parts. Amplification and detection of the error signal of the RFB is done by a conventional dual quadrature lock-in amplifier operated with an external reference signal from the RFB synchronizing-signal generator. A strip chart recorder is used to display the lock-in amplifier output when very low ultrasound levels require visual integration of the output display; otherwise, a critically damped zero-center galvanometer is used. Both the in-phase and quadrature channels are monitored as balancing adjustments are made.

Synchronizing signals for the ultrasound source, the lock-in amplifier, and the nulling pulse generator are provided by the synchronizing-signal generator circuitry of the electronics package. Dual programmable frequency dividers convert a 10 MHz clock signal into two synchronizing signals of identical frequency, adjustable in 0.1 Hz increments near 20 Hz, with phase difference continuously adjustable to 17 ms in 0.1 µs increments. Even in the absence of delays internal to the two electronic systems, a phase difference between the synchronizing signals for the ultrasound source and the nulling pulse generator is needed to compensate for lag. High stability of the frequency and the phase difference of these synchronizing signals is needed to maintain the accuracy of the balance between the radiation-induced and the nulling forces.

Maintaining uniform nulling pulse characteristics for a wide range of ultrasound power levels (0.3 mW to 30 W) imposes special design requirements. Since the voltage applied to the actuator is only a few microvolts at the low end of the RFB operating range, the possibility of interference by induced voltages cannot be dismissed. By deriving the actuator coil voltage from a remotely controlled attenuator mounted as close as possible to the RFB, the length of cable carrying such low voltages is minimized. Further protection is provided by the attenuation imposed by the 45 Ω resistor, located as close as possible to the coil and connected in series with it, which sets the impedance of the actuator to 50 Ω. Degradation of the shape of the nulling pulses by transmission through the coaxial cable which links the nulling pulse generator, attenuator, and actuator is minimal because the impedances of all three devices match the 50 Ω impedance of the cable. Constructed of inexpensive deposited-film attenuator modules switched by mercury-wetted relays, the attenuator is extensively shielded against electromagnetic interference. Protection from drift consequent to internal heating is promoted by circuitry which minimizes the power applied to the relay coils.

### 3.1 Nulling Pulse Generator

Pulses of electric current for the actuator, which generates the nulling force, are provided by the nulling pulse generator circuitry of the electronics package. Key features of this circuit minimize the rise and fall times of the nulling pulses, which are rectangular in waveform, and provide tight control of their amplitude and duration. Provision for adjustment of nulling pulse duration and amplitude allows the envelope of the pulses of nulling force to be made to match that of the pulses of radiation force encountered when measuring the output of any ultrasound system. Matching the envelopes obviates corrections which would require detailed analysis of the behavior of the target and armature as a ballistic pendulum. Because proof test results have established that equal-area rectangular nulling pulses of duration ranging from 100 µs to 800 µs induce indistinguishably different amplitudes of armature motion, the nulling pulse generator is not equipped for fine adjustment of duration. Provision is made for fixed durations of 100 µs, 200 µs, 400 µs, and 800 µs, and for an adjustable duration of approximately 25 ms. Used when testing ultrasound sources operated at high duty factors, the adjustable duration is set to exactly half the period of motion of the RFB target.

Special circuitry allows the nulling pulse amplitude to be inferred from measurements of a direct current seamlessly switched between the actuator coil and a dummy load.

### 3.2 Attenuator

**Fig. 3:**
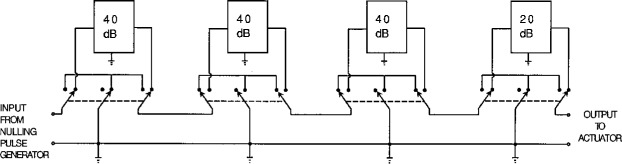
Attenuator circuit (60 dB shown).

As is shown schematically in Fig. 3, the attenuator consists of three 40 dB sections and one 20 dB section in cascade. Each section uses a 50 Ω monolithic metal film attenuator module rated for use at frequencies up to 1.5 GHz. Mercury-wetted relays bypass or insert various sections as needed to obtain nominal total attenuations from 0 dB to 140 db in 20 dB steps.

### 3.3 Auxiliary Electronics

**Fig. 4:**
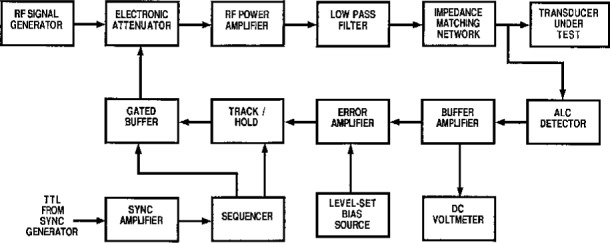
Auxiliary electronics for transducer calibrations.

Many requests for RFB services involve the calibration of transducers intended for use as ultrasound power transfer standards. In such cases both the ultrasound power output and some indicator of the amplitude of the radio-frequency (rf) voltage input are measured simultaneously. Attainment of the best accuracy possible in these transfer calibrations requires measurement and control of the rf drive voltage to within 0.1 % of its mean value. Use of a NIST Standard Ultrasonic Source designed for this purpose demands that this level of rf voltage stability be maintained even when tone burst duty factor changes between 50 % and 100 %. Perhaps because most rf applications require voltage stability no better than a few percent, readily available equipment is inadequate for use as the rf source for transfer calibrations. Accordingly, the commercially available and purpose-built equipment shown in Fig. 4 was assembled. A test-bench synthesized rf signal generator, its output gated by an inexpensive electronic attenuator module, drives a commercial linear rf power amplifier whose output harmonic content is reduced by a lowpass filter of conventional design [24]. Connected directly to the terminals of the transducer, the automatic level control (ALC) sampler module contains an envelope detector, with total shunt capacitance held to 1 pF to avoid waveform distortion by detector diode conduction. Although the dynamic range of a simple diode detector is small, it is much greater than the amplitude increment to be maintained. Built around conventional op amps, the stabilizer uses appropriately sequenced gating to overcome the slow response of the detector consequent to its unusually high impedance.

## 4. Operating Procedure

Ultrasound power measurements are made by manual adjustment of the amplitude and phase of the nulling signal to minimize the RFB error signal. Automation of the process has not been pursued because the effort expended to operate the RFB manually is minimal compared to the level of effort that would be required to program the diversity of strategies dictated by the wide measurement range of the RFB.

Removal of the RFB from standby status is begun by applying ac power to all the electronics of the RFB and the ultrasound source. During the half-hour required for equipment warmup, the test tank, with the transducer already installed and sealed, is set into place on the RFB table. Next, the target is mounted on the armature shaft. All cables are connected as required, and mechanically anchored to the isolated platform under the RFB and to the adjacent tabletop. By carefully arranging the cables to maximize the length and flexibility of the sections linking the anchor points, vibration transmission to the platform through the cables is minimized. Fiducial marks on the test tank allow it to be centered under the tip of the target within 1 mm of the ideal position. Degassed, distilled water is then poured into the tank. Any bubbles created during pouring are swept off the target and secondary absorber surfaces using a rubber-tipped wire. From its initial position at the top of its range of travel, the target is slowly lowered into the water until its tip just touches the face of the transducer, and then is retracted 1.5 mm as indicated on a scale attached to the RFB frame. After a few minutes, a second check for bubbles is undertaken and any new ones are swept out.

Setup of the electronics begins with synchronization of the ultrasound source. Systems designed to generate repetitive pulse sequences need only be connected to the trigger signal from the RFB electronics; all systems of this type tested thus far have been easily and reversibly modified to allow interruption of internal trigger signals. Systems equipped only for continuous wave output are tested by externally switching the transducer drive signal between the transducer and a dummy load. Optical isolation of the RFB trigger signal protects both the NIST and the customer equipment.

After synchronized operation of the ultrasound source has been verified, the nulling pulse duration is set to the shortest of available value that exceeds the duration of the ultrasound pulses to be measured. If not already known, this duration is easily obtained by checking the waveform of either the rf drive to the transducer or the output of a needle hydrophone temporarily inserted into the test tank. Because the mechanical resonance frequency of the RFB is slightly affected by the geometry of the tank and its net volume of water, maximization of error signal sensitivity requires adjustment of the synchronizing-signal frequency every time a test tank is installed. This adjustment is made using a full scale error signal simulated by setting the nulling pulse amplitude higher than would be required for the highest expected level of ultrasound. Using the appropriate lock-in amplifier output, the magnitude of the error signal is measured for the seven synchronizing-signal frequencies, separated by 0.1 Hz, which bound and include the known nominal center frequency for the particular tank in use. Experience has established that the error signal will be maximal at a frequency within this range. All further work is done with the synchronizing-signal frequency set to the optimal value found using this procedure. In order to maximize the sensitivity of the in-phase (I) channel of the lock-in amplifier, its reference channel phase shift is adjusted to force the output of the quadrature-phase (Q) channel to zero. This makes the I channel optimal for use as the RFB error indicator for adjustments of nulling signal amplitude, and the Q channel optimal for adjustments of nulling signal phase.

With nulling pulses off, the ultrasound source is enabled and, if adjustable, set for full power output. Lock-in amplifier phasing is checked and fine tuned as needed. A rough balance is established by switching nulling pulses on and adjusting their amplitude and phase using successively higher lock-in amplifier sensitivity settings, until noise-induced random variation of the error signal exceeds 50 % of the full scale range of the critically damped zero-center galvanometer used for all setup work. This procedure ensures that the optimal lock-in sensitivity setting will be determined unequivocally.

At this point, a last-minute check is made of the $5\frac{1}{2}$ digit voltmeter which indicates nulling signal amplitude. A commercial dc reference standard is set to apply 1 V and then 10 V to the voltmeter. Readings are taken and compared to the ranges allowed by the specifications of the dc standard alone. If either reading falls outside of range, the voltmeter is deemed unfit for RFB service and replaced by an identical one which is then subjected to the same test. After voltmeter performance has been verified, a mercury-in-glass thermometer previously placed in the test tank is removed, quickly read, and the temperature recorded for later use.

Each time a different power level is to be measured, the lock-in amplifier sensitivity is optimized by increasing it until random variations of the I channel output are easily perceptible. Having been established during setup, the nulling pulse phase need be checked only infrequently during a set of measurements because the cosine phase dependence of I channel sensitivity reduces to insignificance the effects of the small deviations typically encountered.

An individual measurement is made by first adjusting the nulling signal amplitude so that all instantaneous values of the I channel output of the lock-in amplifier are well removed, in one direction, from the zero mark on the error signal indicator. This ensures that each measurement will be fully independent of all the others. Next, the nulling signal is adjusted to center the error signal about the zero mark. To further randomize the effects of any bias in operator procedure, successive measurements are made by approaching the null condition in alternating directions, i.e., with the nulling signal too high or too low. Measurements are repeated until the standard deviation for the set, computed as data are taken, stabilizes. Five measurements are sufficient under most circumstances. If the error signal variations reflect low power levels, or unstable output from high power sources, as many as 15 measurements may be made. For each set of measurements, the individual voltmeter readings are recorded and retained permanently. This sequence of operations is repeated for each different power level to be measured.

A zero-center galvanometer is used as the RFB error signal indicator (Fig. 2) for measurements of power levels greater than 1 mW. Otherwise, a strip chart (*x*−*t*) recorder is used to graphically display the error signal so that the RFB operator may visually average the signal even when very long (multisecond) lock-in amplifier time constants are engaged.

Because the RFB design ensures that the characteristics of the actuator and attenuator are not affected by routine measurement operations, these RFB components need not be calibrated for each measurement of ultrasonic power output. Instead, they are calibrated at intervals calculated to minimize the effort required to verify that changes in their characteristics contribute negligibly to the overall RFB measurement uncertainty.

### 4.1 Actuator Calibration

For this operation, the RFB target is replaced by a special pan. To keep the initial position of the armature during calibration the same as during routine power measurements, an adjustable dc current is applied to the velocity sensor coil. Preserving the position of the armature avoids the effects of nonuniformities in the internal magnetic field of the actuator. A built-in quadrature Michelson interferometer, equipped with an electronic bidirectional fringe counter, allows the absolute position of the armature to be determined in increments of 79.1 nm. Calibration of the actuator is begun by adjusting the velocity sensor coil current so that the counter reading fluctuates by no more than one count. Just after this counter reading is logged, a mechanical load in the form of an object of known mass (nominally 10 g) is placed on the pan. This causes the counter reading to change by about 324 counts. Next, an adjustable dc current source, preset to a nominal level, is connected to the actuator coil and readjusted so that the counter reading returns to the number logged with the pan empty. As soon as the actuator coil current has been measured, logged, and the logged value checked against a second measurement, the current source is disconnected. With the pan still loaded, the counter reading is logged for comparison with the reading obtained before the actuator coil current was switched on. If the difference exceeds one count, the procedure is restarted. After the pan has been unloaded, the counter reading is logged for comparison with the original reading with the pan unloaded. If this difference exceeds one count, the measured value of actuator coil current is deemed an outlier and discarded. This sequence of operations is repeated until 30 measurements of actuator coil current have been accumulated. At the conclusion of this exercise, the resistances of the actuator coil and an auxiliary resistor for monitoring actuator coil current are each measured using an ohmmeter with Kelvin terminal connections.

### 4.2 Attenuator Calibration

In order to minimize concomitant uncertainties, the attenuator is characterized by the ratio which results from dividing the voltage across the actuator terminals by the voltage across a current-sensing resistor in the nulling pulse generator. This method of characterization eliminates both the effort and the additional errors that would be associated with the use of the exact resistances of the sensing resistor and the actuator, compensates for the effects of cable and connector resistances, and allows the nulling pulse generator itself to serve as the stable dc source for these tests.

Exact values are computed by averaging five ratios derived from voltages set to approximately 50 %, 60 %, 65 %, 70 %, and 75 % of the full scale readings of the identical $5\frac{1}{2}$ digit instruments used to measure these voltages. This averaging scheme minimizes the effects of voltmeter nonlinearities, and is also used for the intermediate ratios needed to calibrate the attenuator settings for which one-step computations are impractical because of the inaccuracies of measuring excessively low voltage, and the thermal considerations which preclude applying excessively high voltage to the input of the attenuator. Intermediate ratios are computed from voltages measured at the input and output of each section for all settings which load the section differently. With interconnection losses automatically taken into account by the locations of the test points, this procedure compensates for the effects of variations in loading due to the inevitable slight differences in input and output resistances of the four sections.

## 5. Measurement Uncertainty

Since 1992 it has been NIST policy to express the uncertainty of measurements made at NIST in conformance with the approach recommended by the International Committee for Weights and Measures (CIPM). As interpreted [25], this approach requires each component of uncertainty to be described in terms of the standard uncertainty defined by the positive square root of the variance of the applicable set of measurements. For each measurement result, a combined standard uncertainty is determined by taking the square root of the sum of the squares of the individual standard uncertainties. An expanded uncertainty, determined by multiplying the combined standard uncertainty by a coverage factor, is used to define the confidence interval for each measurement result. For consistency with current international practice, the value used for the coverage factor is 2. Components of uncertainty whose numerical values are evaluated by statistical means are designated Type A, and components of uncertainty whose numerical values are evaluated by other means are designated Type B.

Hereinafter, the word uncertainty is used as shorthand for relative standard uncertainty expressed as a percentage of the mean of the values composing the applicable data set. Uncertainties derived from statistical analysis of the results of performance tests of RFB equipment are designated Type A. Uncertainties taken from equipment manufacturers’ specifications are designated Type B.

### 5.1 Measurement Uncertainty—Instrumental

Levels of ultrasound power measured using the RFB are subject to the usual effects of imperfect operation of equipment, and are also affected by the subtleties of ultrasonic wave propagation within the RFB itself. It is convenient to consider the instrumental effects separately from the phenomenological ones.

Throughout this analysis, component uncertainties less than 0.005 % are rounded up to 0.01 %, and the rest are rounded to the nearest 0.01 % for computational use. Simple translation of manufacturer’s specifications is used to quantify uncertainty components no greater than 0.03 %, since the combined standard uncertainty for the RFB is known to be at least ten times larger. Components whose magnitude exceeds 0.03 % are statistically extracted from experimental data. When only the limiting values of a design parameter are known, a purposefully conservative approach is taken—the standard uncertainty is reckoned, without regard to ramifications of possible underlying statistical distributions, to be the larger difference between a limiting value and the nominal value. For clarity, uncertainties are grouped according to terms of equations describing RFB operation, rather than by their Type A and Type B designations.

For obvious practical reasons, it is desirable to measure the total power *P* radiated from the output port of a transducer, rather than the power *W* intercepted by the RFB target. By assuming that the two differ by an empirically derived attenuation correction factor *C*, recasting Eq. (1), and invoking Newton’s First Law, the radiated power *P* is given by

P=VacCGKg (2)

where *V* is the average of a set of voltmeter readings for a particular power level, *A* is the attenuator coefficient for the attenuator configuration in use, *c* is the speed of sound in water at the time of the measurement, *C* is the attenuation correction factor applicable to the transducer under test, *G* is the actuator conductance, *K* is the actuator calibration coefficient, and *g* is the local acceleration of gravity. From left to right, the variables are listed in decreasing order of frequency of revision during a typical RFB work session.

Average values *V* of the voltmeter readings recorded for each power measurement are affected by both the 0.01 % Type B uncertainty derived from the meter manufacturer’s specifications and by various physical phenomena which manifest themselves in the 0.20 % Type A uncertainty that is typical for a set of five successive voltmeter readings.

Attenuator coefficient *A* is determined by a single voltage ratio for the highest power ranges of the RFB, and by the product of as many as four ratios for the other ranges. Since the three most commonly used ranges involve values of *A* derived from no more than two ratios, that case will be taken as typical. Each ratio is calculated by averaging the quotients of five pairs of voltage measurements. Over the range of voltages measured in determining *A*, the Type B uncertainty derived from the meter manufacturer’s specifications is 0.01 %. By combining the 0.02 % Type A uncertainties for each ratio, and allowing for 20 voltmeter readings, the typical uncertainty applicable to *A* is found to be 0.05 %.

Values of *c*, the speed of sound in water, are obtained from fitted data tabulated in increments of 0.1 °C [23]. By using a thermometer of the same resolution to determine the water temperature, the worst case (Type B) uncertainty in *c* is held to 0.03 % for temperatures near the laboratory ambient.

Actuator conductance *G* is calculated by averaging the results of four ohmmeter readings made using Kelvin terminal connections. When taking a set of four readings, no reading has ever differed from the mean by more than 0.004 %. Under this circumstance, the uncertainty of values of *G* is taken to be the 0.02 % Type B uncertainty derived from the ohmmeter manufacturer’s specifications.

Actuator coefficient *K* is the product of measured values of mass and electrical resistance, divided by an average measured voltage. Testing by the NIST Mass Group allowed the mass to be determined with 0.0002 % Type A uncertainty. According to the multimeter manufacturer’s specifications, the Type B uncertainties applicable to the measured values of resistance and voltage are 0.03 % and 0.01 %. By combining these three uncertainties and the 0.15 % Type A uncertainty which characterizes a typical set of 30 voltage measurements, the uncertainty applicable to *K* is found to be 0.15 %.

Derived from measurements of the acceleration of gravity at various locations on the NIST campus, the last term of the equation is deemed constant because the measured range did not exceed 0.003 % of the mean [26]. This term contributes 0.01 % to Type B uncertainties summarized later.

By combining the uncertainties derived for these six terms of Eq. (2), the combined uncertainty for instrument readings under typical conditions is found to be 0.26 %. Of this, 0.25 % corresponds to combined Type A uncertainty and 0.07 % correspond to combined Type B uncertainty.

### 5.2 Measurement Uncertainty—Phenomenological

Additional difficulties, potentially more significant than those just addressed, can arise from the imperfect performance of the RFB target as a power-to-force transducer, and from phenomena which affect the ultrasound beam along its short path from transducer to target.

For a conical target of half-angle *ϕ*, a sound beam, with propagation axis parallel to that of the cone, induces a force which differs from the ideal radiation force by the factor (1 − *β* ^2^ cos 2 *ϕ*), where *β* is the amplitude reflection coefficient [5]. If *ϕ* is exactly 45°, the design value for the RFB target, then the cosine term and the consequent error will be zero regardless of the value of *β*. Construction of the RFB target was done using techniques likely to allow *ϕ* to differ from 45° by no more than 1°. Independent measurements, made using samples of the silicone rubber used to construct the target established the value of *β* to be 0.16. Taking these data into account, a Type B uncertainty of 0.09 % is attributed to possible imperfections in the shape of the target.

Further difficulties can arise from misalignment of the sound beam, target, and RFB armature. Under ideal circumstances, the axes of all three lie on the same straight line. If the axes are instead merely parallel, no significant error will arise so long as the target intercepts the entire sound beam. Similarly, with the axes of the sound beam and RFB parallel, misalignment of the axis of the target will not affect the magnitude of the force induced in the direction of the beam. Lack of parallelism between the axes of the sound beam and the RFB armature will introduce a cosine error. For the anticipated worst case 2° angular error, the consequent Type B uncertainty is 0.06 %.

In order to determine the output of the ultrasound source being tested, as distinct from the input to the RFB target, the attenuation due to the water in between must be taken into account. This task is complicated by the fact that ultrasound rays which emanate perpendicularly from all locations on the face of an unfocused transducer must travel different distances to reach the surface of the conical target. For a circular ultrasound beam centered on a conical target whose half-angle is 45°, the normal ray from the transducer to the vertex of the cone travels 1.5 mm, the separation established during setup, and the rays to the edge of the cone travel a distance longer by the radius of the transducer. Since most ultrasound sources tested using the RFB are at least several millimeters in radius, the variations in path length are significant.

Under realistic conditions in which the ultrasound beam is circularly symmetric and the water underneath the target is homogeneous in its attenuative properties, the total radiation force generated by the conical target will be the same as that generated by a flat target located some distance farther away from the ultrasound source. This distance defines the effective entrance plane of the RFB, is a function of both the radius of the ultrasound beam and the level of attenuation in the water, and can be determined by summing the forces induced on a set of elementary rings composing the cross section of the conical target. Because large ultrasound beams are typically characterized by low amplitudes and frequencies only slightly attenuated in water, the uncertainty in location of the entrance plane adds no more than 0.01 % of Type B uncertainty to measurements of ultrasound power.

Under idealized circumstances, both the radiation pressure and resultant force on a target removed a distance *x* from an ultrasound source would change by the factor exp(−2*αx*), where *α* describes the attenuation in the surrounding medium. However, the induced force can change by a lesser amount if some of the ultrasound energy lost to attenuation is translated into momentum, in effect inducing a radiation force on the water itself. With the attenuation in water virtually nil at the audio frequencies used to modulate the ultrasound measured by the RFB, any vibratory momentum imparted to the water under the target would induce on the target a force indistinguishable from the modulated force induced by the ultrasound, partially counteracting the attenuation intrinsic to the water and precluding the use of handbook values in correcting for attenuation in the RFB.

Because the RFB is equipped for continuous adjustment of the distance between the source and the target, attenuation in the water under the target is easily determined *in situ* by measuring the output of a source for a number of different distances and averaging the ratios of measured values corresponding to successive increments. Using a transducer capable of operation at harmonic frequencies allows the frequency dependence of the *in situ* attenuation to be determined without changing other systematic variables. Data for the frequency range 2 MHz to 31 MHz show that effective attenuation in the RFB differs significantly from that predicted using a handbook [27] value, 24×10^−15^ s^2^·m^−1^, for the attenuation coefficient of water. Ranging from 0.006 dB/mm at 2 MHz to 0.07 dB/mm at 31 MHz for a transducer 16 mm in diameter, the differences correspond to discrepancies ranging from 1.1 % to 5.5 % in values of the output power of typically sized transducers after compensating for attenuation using handbook values. Unacceptably large, these discrepancies are avoided by using each ultrasound source submitted for testing to determine the *in situ* values of attenuation applicable to all conditions under which it is tested, and then using only those values in correcting for attenuation. This procedure contributes 0.56 % of Type A uncertainty to reported values of power.

Taking the effects of target shape, alignment, entrance plane location, and attenuation into account, the phenomenological uncertainty typically applicable to RFB power measurements is found to be 0.57 %. Of this, 0.56 % corresponds to Type A uncertainty, and 0.11 % corresponds to Type B uncertainty.

### 5.3 Overall Measurement Uncertainty

Unaffected by the circumstances of a particular power measurement, the uncertainties attributable to the acceleration of gravity, the speed of sound in water, the actuator calibration coefficient, the attenuator coefficient, the actuator coil conductance, and the target shape, alignment, and entrance plane location, constitute the general uncertainty of RFB power measurements. By combining the values listed for these eight components, the general uncertainty of RFB power measurements is found to be 0.20 %. Of this, 0.15 % corresponds to Type A uncertainty, and 0.13 % corresponds to Type B uncertainty.

An uncertainty specific to each power measurement is determined by combining this general uncertainty with the uncertainties due to the voltmeter readings which determine *V* for each power measurement, and the data from which *in situ* attenuation is determined for the device under test. For ultrasound sources of typical stability generating at least a few milliwatts, these Type A uncertainties amount to approximately 0.20 % and 0.56 %, respectively. By combining these with the 0.20 % general uncertainty just derived, the uncertainty specific to a typical test is found to be approximately 0.63 %. Of this, 0.61 % corresponds to Type A uncertainty, and 0.13 % corresponds to Type B uncertainty.

Much higher uncertainties have been found to apply to the results of measurements made under atypical conditions. With low-power diagnostic equipment whose output is not much above the noise floor, and high-power therapeutic equipment whose output is subject to fluctuation, combined standard uncertainties in excess of 7 % have been found.

All reports of the results of RFB measurements include, for each measured value of ultrasound power, a corresponding value of expanded uncertainty [28] obtained using two as the coverage factor by which the specific combined standard uncertainty is multiplied.

## 6. Conclusion—Ultrasound Measurement Capabilities

Ultrasonic power levels ranging from 100 µW to 30 W at frequencies between 0.5 MHz and 30 MHz can be measured using the NIST RFB. Circular transducers of any diameter smaller than 50 mm can be accommodated routinely; other shapes and sizes may be tested by special arrangement. Spatial-average-temporal-average power output can be measured for any ultrasonic system capable of synchronizing its output pulse train with an external signal of nominal frequency 20 Hz.
